# Are circumcised men safer sex partners? Findings from the HAALSI cohort in rural South Africa

**DOI:** 10.1371/journal.pone.0201445

**Published:** 2018-08-01

**Authors:** Molly S. Rosenberg, Francesc X. Gómez-Olivé, Julia K. Rohr, Kathleen Kahn, Till W. Bärnighausen

**Affiliations:** 1 Department of Epidemiology and Biostatistics, Indiana University School of Public Health-Bloomington, Bloomington, Indiana, United States of America; 2 Center for Population and Development Studies, Harvard University, Cambridge, Massachusetts, United States of America; 3 MRC/Wits Rural Public Health and Health Transitions Research Unit (Agincourt), School of Public Health, Faculty of Health Sciences, University of the Witwatersrand, Johannesburg, South Africa; 4 INDEPTH Network, Accra, Ghana; 5 Umeå Centre for Global Health Research, Division of Epidemiology and Global Health, Department of Public Health and Clinical Medicine, Umeå University, Umeå, Sweden; 6 Africa Health Research Institute, Somkhele, KwaZulu-Natal, South Africa; 7 Institute of Public Health, Faculty of Medicine, University of Heidelberg, Heidelberg, Germany; Cardiff University, UNITED KINGDOM

## Abstract

**Introduction:**

The real-world association between male circumcision and HIV status has important implications for policy and intervention practice. For instance, women may assume that circumcised men are safer sex partners than non-circumcised men and adjust sexual partnering and behavior according to these beliefs. Voluntary medical male circumcision (VMMC) is highly efficacious in preventing HIV acquisition in men and this biological efficacy should lead to a negative association between circumcision and HIV. However, behavioral factors such as differential selection into circumcision based on current HIV status or factors associated with future HIV status could reverse the association. Here, we examine how HIV prevalence differs by circumcision status in older adult men in a rural South African community, a non-experimental setting in a time of expanding VMMC access.

**Methods:**

We analyzed data collected from a population-based sample of 2345 men aged 40 years and older in a rural community served by the Agincourt Health and socio-Demographic Surveillance System site in Mpumalanga province, South Africa. We describe circumcision prevalence and estimate the association between circumcision and laboratory-confirmed HIV status with log-binomial regression models.

**Results:**

One quarter of older men reported circumcision, with slightly more initiation-based circumcisions (56%) than hospital-based circumcisions (44%). Overall, the evidence did not suggest differences in HIV prevalence between circumcised and uncircumcised men; however, those who reported hospital-based circumcision were more likely to test HIV-positive [PR (95% CI): 1.28 (1.03, 1.59)] while those who reported initiation-based circumcision were less likely to test HIV-positive [PR (95% CI): 0.68 (0.51, 0.90)]. Effects were attenuated, but not reversed after adjustment for key covariates.

**Conclusions:**

Medically circumcised older men in a rural South African community had higher HIV prevalence than uncircumcised men, suggesting that the effect of selection into circumcision may be stronger than the biological efficacy of circumcision in preventing HIV acquisition. The impression given from circumcision policy and dissemination of prior trial findings that those who are circumcised are safer sex partners may be incorrect in this age group and needs to be countered by interventions, such as educational campaigns.

## Introduction

Voluntary medical male circumcision (VMMC) is highly efficacious in preventing HIV acquisition in men.[1–4] In South Africa, where HIV incidence has been extremely high over the past three decades, national guidelines recommend that all men who test HIV-negative be offered a comprehensive prevention package that includes information on and referral to VMMC services, and that “the success of testing programs should be measured by the number of individuals successfully linked to…medical male circumcision services for HIV-negative males.”[5]

However, little is known about the current differences in HIV prevalence by circumcision status in real-world, non-clinical trial settings. The evidence justifying VMMC trials came from a body of observational studies demonstrating lower HIV prevalence among traditionally circumcised men.[6] It is unknown if this association differs after years of expanding VMMC access when the decision to undergo the procedure may relate to current HIV status or factors associated with future HIV status, and specifically whether these kinds of selection effects could be strong enough to outweigh the protective biological effect of VMMC. One message transmitted by current VMMC policy and the proven protective effect of VMMC is that circumcised men are less likely to be HIV-positive. There is emerging potential for female partners, who report preference for circumcision in part due to perceptions about HIV status,[7,8] to change their sexual behaviors in response to this assumption.[9,10] The implications for HIV risk rest on whether or not this assumption is founded.

There is also little known about male circumcision acceptability and uptake among older men in South Africa. In younger and general population South African and sub-Saharan African men, circumcision is generally perceived positively, with the link between circumcision and HIV/STI prevention cited as an important reason to become circumcised.[11–13] Older men, who are often overlooked as targets for HIV prevention interventions due to incorrect assumptions about their lack of sexual risk-taking,[14,15] and who came of age prior to VMMC recommendations for HIV prevention, may have different circumcision attitudes and patterns of circumcision uptake. Further, circumcision associated with traditional initiation ceremonies marking the rite of passage from childhood to adulthood is relatively common among Black South Africans and is more prevalent among older men than younger men.[16]

In this paper, we aim to fill persistent knowledge gaps related to male circumcision in older men in sub-Saharan Africa. First, we describe the prevalence of male circumcision in the population and identify socio-demographic predictors of being circumcised in a sample of men 40 years and older from a rural South African community. Then, we model the association between circumcision status and prevalent HIV infection to provide insight into how HIV prevalence differs by circumcision status in a non-experimental setting in a time of expanding VMMC access.

## Methods

### Study population

This analysis uses data from the population-based Health and Aging in Africa: A Longitudinal Study of an INDEPTH Community in South Africa (HAALSI) study that aims to characterize a community of older men and women in rural South Africa with respect to health, physical and cognitive function, aging, and well-being. Adults age 40 and older were sampled from the existing framework of the Agincourt Health and Socio-Demographic Surveillance System (Agincourt HDSS)[17] in Mpumalanga province, northeast South Africa, and visited at home between November 2014 and November 2015. Extensive survey and laboratory data were collected to assess physical and cognitive functioning, cardiometabolic health, economic well-being, and HIV and HIV risk.

Ethical approval for HAALSI was obtained from the University of the Witwatersrand Human Research Ethics Committee (#M141159), the Harvard T.H. Chan School of Public Health Office of Human Research Administration (#13–1608), and the Mpumalanga Provincial Research and Ethics Committee. All participants provided written informed consent to participate in the HAALSI study with consent procedures approved by the three ethical committees mentioned above.

### Key measures

Trained, local fieldworkers collected survey data electronically using Computer Assisted Personal Interviews (CAPI). Surveys were conducted in the local language, Shangaan, with instruments translated from English and back-translated to ensure accurate translation. *Circumcision status* was ascertained with the question: “Has your foreskin been removed?” Among those who responded affirmatively to the circumcision question, *age at circumcision* (ages 0–13 14–18, and 19+) and *type of circumcision* (hospital-based or initiation-based circumcision) were queried. Although an ‘other’ response option for type of circumcision was available, all circumcised men self-selected into either ‘initiation’ or ‘hospital’ categories. For this reason, these are the only categories we used.

In addition to the survey, fieldworkers collected blood via finger prick from those who consented. Dried bloodspots were prepared and later tested for HIV. *Laboratory-confirmed HIV status* was determined through screening and confirmatory HIV enzyme-linked immunosorbent assays conducted using standard laboratory practices on the prepared dried bloodspots.[18]

### Statistical analysis

Given the proven biological efficacy of VMMC, we hypothesized that circumcised men would have lower HIV prevalence than uncircumcised men, and that lower HIV prevalence would be explained by those receiving hospital-based circumcisions and circumcisions at younger ages. We used log-binomial regression models to estimate the association between circumcision status and HIV status, with circumcision status stratified by age at circumcision and type of circumcision, and then doubly stratified by age and type of circumcision. We chose log-binomial regression models over logistic regression models because prevalence ratios from log-binomial regression are generally more interpretable than odds ratios from logistic regression. As a sensitivity analysis around our modeling decisions, we repeated our analyses using logistic regression models.

We compared unadjusted estimates to estimates adjusted for age, in years; household asset index quintiles; African traditional religion; marital status; country of origin; formal education (any versus none); and number of lifetime sex partners (0–1, 2–4, or 5+). This study does not seek to re-examine the established causal relationship between VMMC and reduced HIV risk. Instead, we were interested in how circumcision and HIV prevalence are associated in a real-world setting of older men, in which decisions to undergo circumcision procedures could plausibly be related to HIV status and/or HIV risk. Thus, the unadjusted analyses of the association between circumcision and HIV are the primary results of interest in this study. The adjusted analyses provide evidence for whether these population-level estimates can be explained by the inclusion of key covariates. All analyses were conducted using SAS statistical software, version 9.4.[19]

## Results

Of the 2345 men enrolled in the study, 68% were currently married, 41% reported no formal education, 11% reported African/traditional religious affiliation, and 29% were of Mozambican descent (Table 1). Median age was 61 years with interquartile range between 52 and 71 years.

**Table 1:** Sociodemographic characteristics of HAALSI men, by circumcision status and circumcision type.

	Total men (n = 2345)		Uncircumcised^1^ (n = 1609)		Hospital-based circumcision^1^ (n = 244)		Traditional initiation-based circumcision^1^ (n = 308)		
	N	%	N	%	N	%	N	%	p
**Age**									<0.0001
40–49	418	17.8	270	16.8	79	32.4	42	13.6	
50–59	624	26.6	434	27.0	69	28.3	83	27.0	
60–69	643	27.4	450	28.0	59	24.2	89	28.9	
70–79	446	19.0	311	19.3	25	10.3	62	20.1	
80+	214	9.1	144	9.0	12	4.9	32	10.4	
**Marital status**									0.006
Never married	166	7.1	101	6.3	19	7.8	30	9.7	
Separated/divorced	300	12.8	194	12.1	39	16.0	48	15.6	
Widowed	276	11.8	203	12.6	16	6.6	40	13.0	
Currently married/cohabitating	1602	68.3	1111	69.1	170	69.7	190	61.7	
**Educational attainment**									<0.0001
No formal education	957	40.9	726	45.2	46	18.9	93	30.2	
Some primary (1–7 years)	819	35.0	546	34.0	85	34.8	136	44.2	
Some secondary (8–11 years)	303	13.0	187	11.6	48	19.7	50	16.2	
Secondary or more (12+ years)	259	11.1	148	9.2	65	26.6	29	9.4	
Missing	7		2		0		0		
**Religion**									0.007
None	696	29.7	481	30.0	59	24.2	97	31.5	
Christianity and Islam^2^	1384	59.1	934	58.2	170	69.7	178	57.8	
African traditional	262	11.2	191	11.9	15	6.2	33	10.7	
**Country of origin**									<0.0001
South Africa	1663	70.9	1046	65.0	212	86.9	273	88.6	
Mozambique/Other	682	29.1	563	35.0	32	13.1	35	11.4	
**Wealth index quintiles**									0.0002
Q1	475	20.3	332	20.6	30	12.3	75	24.4	
Q2	465	19.8	347	21.6	38	15.6	50	16.2	
Q3	458	19.5	323	20.1	48	19.7	58	18.8	
Q4	434	18.5	280	17.4	51	20.9	55	17.9	
Q5	513	21.9	327	20.3	77	31.6	70	22.7	
**Number of lifetime sex partners**									0.003
0–1	215	10.8	174	12.2	23	8.7	9	4.3	
2–4	658	32.9	479	33.7	86	32.5	65	31.1	
5+	1126	56.3	769	54.1	156	58.9	135	64.6	
Missing	346		187		43		35		
**Age at circumcision**									<0.0001
1–13	-	-	-	-	37	15.3	86	28.1	
14–18	-	-	-	-	62	25.6	137	44.8	
>18	-	-	-	-	143	59.1	83	27.1	
Missing	-	-	-	-	2		2		

^1^Circumcision status was missing for n = 183 men. Of the n = 553 men reporting circumcision, most reported initiation-based ceremony circumcisions (n = 308) or hospital-based circumcisions (n = 244). Only one participant reported ‘other’ for type of circumcision; this observation is not included in this table.

^2^Very few participants in the sample identified as Muslim (n = 2).

One quarter of the men reported circumcision; of them, over half (56%) were circumcised in traditional initiation-based circumcision ceremonies while 44% were circumcised in hospital-based procedures. Those who reported hospital-based circumcision were younger, less likely to be widowed, more likely to have higher educational attainment, more likely to identify with Christian or Islamic religion, less likely to be of Mozambican descent, and more likely to live in a household in the highest wealth quintile, compared to those who were uncircumcised. Those who reported initiation-based circumcision were older, less likely to be married, more likely to have higher educational attainment, and less likely to be of Mozambican descent, compared to those who were uncircumcised.

Nearly a quarter of circumcised men (23%) were circumcised in childhood or early adolescence (before age 14), 36% were circumcised during adolescence (age 14–18), and 41% were circumcised in adulthood (age 19 or older). As expected, those who reported hospital-based circumcisions were more likely to report circumcision in adulthood compared to those who reported initiation-based circumcision.

**Fig 1:**
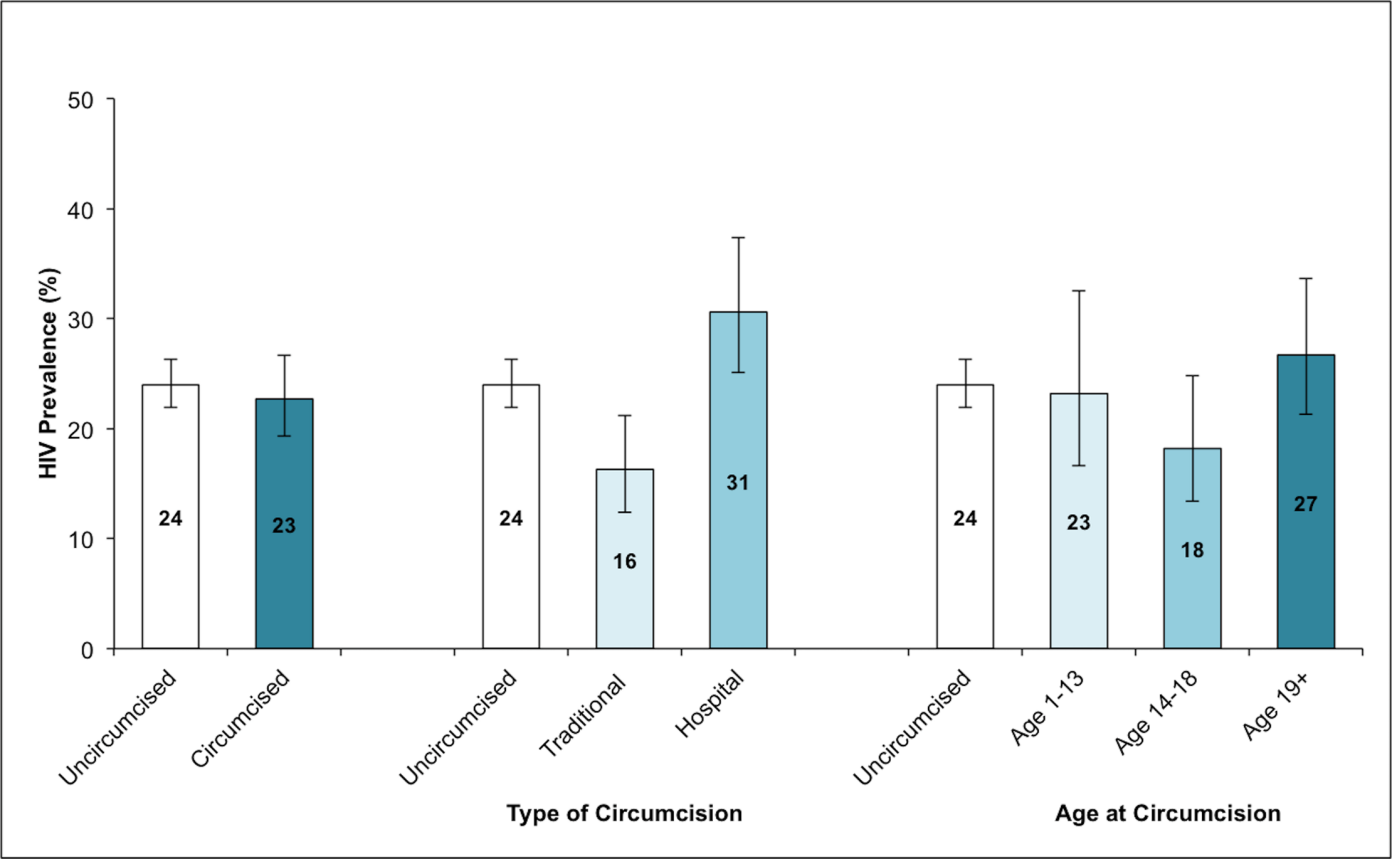
HIV prevalence by circumcision status, circumcision type, and age at circumcision among HAALSI men with laboratory-confirmed HIV status (n = 1945).

Overall, laboratory-confirmed HIV prevalence among HAALSI men was 23% (95% CI: 21, 25), with no evidence for differences between uncircumcised men [24% (95% CI: 22, 26)] and circumcised men [23% (95% CI: 19, 27)] (Table 2 and Fig 1). HIV prevalence was lowest among those who reported an initiation-based circumcision [16% (95% CI: 12, 21)], higher among those who did not report circumcision [24% (95% CI: 22, 26)], and highest among those who reported a hospital-based circumcision [31% (95% CI: 25, 37)]. After adjustment, a protective association between traditional circumcision and HIV prevalence remained [PR (95% CI): 0.73 (0.54, 0.98)], and the association between hospital-based circumcision and heightened HIV prevalence was smaller in magnitude [PR (95% CI): 1.11 (0.88, 1.40)] with confidence interval spanning the null. No clear pattern emerged in HIV prevalence by age at circumcision in adjusted or unadjusted analyses; however, those who were circumcised between the ages of 14 to 18 years were the least likely to test HIV-positive [18% (95% CI: 13, 25)], compared to those who were circumcised at age 13 years or younger [23% (95% CI: 17, 33)], and those who were circumcised after age 18 [27% (95% CI: 21, 34)]. HIV prevalence did not vary significantly by age at circumcision when stratified by circumcision type.

**Table 2:** Relationship between circumcision status and HIV status among HAALSI men with laboratory-confirmed HIV status (n = 1945).

	N	# HIV+	HIV prevalence (95% CI)	PR (95% CI)	p	aPR^1^ (95% CI)	p
**Circumcision status**							
Circumcised	497	113	22.7% (19.3, 26.7)	0.95 (0.79, 1.14)	0.6	0.92 (0.76, 1.12)	0.4
Uncircumcised	1448	347	24.0% (21.9, 26.3)	1		1	
**Circumcision type**							
Hospital-based circumcision	219	67	30.6% (25.1, 37.4)	1.28 (1.03, 1.59)	0.03	1.11 (0.88, 1.40)	0.4
Traditional initiation-based circumcision	277	45	16.3% (12.4, 21.2)	0.68 (0.51, 0.90)	0.007	0.73 (0.54, 0.98)	0.03
No circumcision	1448	347	24.0% (21.9, 26.3)	1		1	
**Age at circumcision**							
1–13	112	26	23.2% (16.6, 32.5)	0.97 (0.68, 1.37)	0.9	0.93 (0.63, 1.36)	0.7
14–18	181	33	18.2% (13.4, 24.8)	0.76 (0.55, 1.05)	0.1	0.74 (0.52, 1.04)	0.09
>18	202	54	26.7% (21.3, 33.6)	1.12 (0.87, 1.43)	0.4	1.07 (0.83, 1.36)	0.6
No circumcision	1448	347	24.0% (21.9, 26.3)	1		1	
**Age at circumcision among hospital-based circumcisions**							
1–13	34	10	29.4 (17.5, 49.5)	1		*	
14–18	55	16	29.1 (19.3, 44.0)	0.99 (0.51, 1.92)	1.0	*	
>18	128	41	32.0 (24.9, 41.2)	1.09 (0.61, 1.94)	0.8	*	
**Age at circumcision among traditional initiation-based circumcisions**							
1–13	77	15	19.5 (12.4, 30.7)	1		*	
14–18	126	17	13.5 (8.7, 21.0)	0.69 (0.37, 1.31)	0.3	*	
>18	74	13	17.6 (10.7, 28.8)	0.90 (0.46, 1.76)	0.8	*	

^1^Adjusted for age (coded linearly in years), socio-economic quintiles, religion (African traditional vs. not), marital status (currently married vs. not), country of origin, education (any formal education vs. none), and number of lifetime sex partners (coded as 0–1, 2–4, or 5+)

*Adjusted analyses not conducted for age at circumcision stratified by circumcision type because data too sparse to support the models (i.e. the number of total HIV outcome events was less than 10 per predictor variable to be included in the adjusted model).

### Sensitivity analysis

Because nearly all Muslims are circumcised for religious reasons, we excluded the small Muslim population (n = 2) from the sample and repeated our analyses. As expected, all of our results remained nearly identical to the main analyses shown in the paper. Odds ratios from logistic regression models were similar in magnitude and direction to the prevalence ratios calculated in our primary analysis (S1 Table).

## Discussion

In this analysis of population-based data on older men in rural South Africa, we examined circumcision history and the association between circumcision and HIV status in the current circumcision landscape. We found that one quarter of older men reported being circumcised, with slightly more than half of circumcisions reported as traditional initiation-based procedures as opposed to medical procedures. This circumcision prevalence is lower than the 2012 national estimate of 46% in men age 15 and older, but in line with findings indicating traditional circumcisions are more common than medical circumcision.[20] We also found that the demographic profile of those who were uncircumcised differed substantially from those with hospital-based circumcisions and those with initiation-based circumcisions. We found no differences in HIV prevalence by circumcision status overall, and that men with hospital-based circumcisions were more likely to test HIV-positive compared to both uncircumcised men and men with traditional initiation-based circumcision.

That hospital-based circumcision was not associated with lower HIV prevalence differs from the findings we expected based on (i) the national policy on selection of HIV-negative men for VMMC, (ii) the protective effect of VMMC on HIV acquisition, and (iii) the protective association we observed between traditional initiation-based circumcision and HIV. A large body of observational studies from the pre-VMMC era established that traditionally circumcised men were less likely to be HIV-positive. A systematic review of 27 studies[6] reported a protective effect in nearly all of the unadjusted analyses, with strengthened associations after covariate adjustment. In population-based, cross-sectional studies like ours, the overall protective effect was 0.55 (95% CI: 0.34, 0.54), slightly stronger than our estimate for traditionally circumcised men, but in contrast to our estimate for medically circumcised men.

Of course, our findings are plausible despite the high biological efficacy of medical circumcision in preventing HIV acquisition in men. The most likely explanation is that HIV-positive older men are circumcised in clinical settings in larger numbers than HIV-negative men, counter to the South African policy to target HIV-negative men with circumcision information and referral. One plausible pathway for this explanation is that HIV-positive men are more engaged in care than their HIV-negative counterparts, and circumcision services could be one of the clinical services to which they have more access.

Another possible explanation is that men who engage in riskier sexual behavior self-select into VMMC, resulting in heightened risk for subsequent HIV infection. Although no significant differences were observed by age at circumcision, HIV prevalence was highest among men who were circumcised in the oldest age category (after age 18). One possible explanation for this finding is that in adulthood men with higher than average risk of HIV acquisition chose to be circumcised but that no such selection effect existed in childhood. However, this explanation requires the perhaps improbable assumption that the risk behaviors among the circumcised confer risk at a magnitude able to overcome the otherwise protective circumcision effect.

Similarly, it is possible that older men engage in riskier sexual behavior after circumcision because they know that they are now at substantially reduced risk for HIV acquisition. However, this explanation is unlikely as there has been no evidence for this kind of risk compensation in prior circumcision studies in Africa,[21–26] and the magnitude of risk compensation that would have had to occur in our study population to bias the otherwise protective effect to become a significant association on the other side of the null is likely implausibly large. The possibility also exists for risk compensation in men with traditional initiation-based circumcision if they had been informed of the protective effects of circumcision. While risk compensation is unlikely to be the only explanation of the observed association between circumcision and HIV status in this study, it could have contributed to some extent to our findings, warranting further research on quantifying the level of risk compensation in communities similar to the study community.

Several features of the data structure and quality call for our findings be interpreted with caution. First, we used self-reported circumcision status with no objective examination to confirm true circumcision status, which could lead to biased results if men were unwilling or unable to accurately report their true circumcision status. Second, the data were cross-sectional in design so we were unable to disentangle the temporal relationship between circumcision and HIV status. Plausible bidirectional mechanisms exist and we were not able to assess their relative contributions given the rough categorization of age at circumcision and unknown date of HIV seroconversion. Finally, these data were collected in a sample of older men in a rural South African community. Generalizability of these results to younger men or men in more urban settings may not be possible.

Our findings are important for HIV prevention policy in South Africa for several reasons. First, among older men in rural South Africa, there remain many HIV-negative uncircumcised men who could receive HIV prevention benefits from VMMC as part of a comprehensive HIV prevention package promoting safer sexual practices tailored to the particular needs of older adults. Expansion of VMMC services to target older men with referrals could help address this unmet need. Second, the policy to target HIV-negative men with VMMC is likely not complied with in real-world settings as HIV prevalence was higher among medically circumcised men than among uncircumcised men. It is plausible that this policy, coupled with the widely-known protective effect of VMMC, gives the false impression that circumcised men are safer sex partners. If HIV-positive men actually take up the procedure at high rates, the promotion of VMMC as a procedure for HIV-negative men to prevent HIV may have the unintended consequence of increasing HIV transmission at the population-level. To reduce this risk, modifications of the existing VMMC policies and associated public engagement campaigns should be considered.

## Conclusions

Medically circumcised older men in rural South Africa had higher HIV prevalence than uncircumcised men, despite the biological efficacy of VMMC and the South African policy explicitly targeting HIV-negative men for circumcision. This finding suggests that the association between circumcision and HIV status may be more strongly driven by selection into circumcision than the biological efficacy of circumcision in preventing HIV acquisition. The impression given from circumcision policy and dissemination of prior trial findings that those who are circumcised are safer sex partners may be incorrect in this age group.

## Supporting information

S1 Table Logistic regression sensitivity analysis of the relationship between circumcision status and HIV status among HAALSI men with laboratory-confirmed HIV status (n = 1945). (DOCX)
