# Supplementary material for: Are circumcised men safer sex partners? Findings from the HAALSI cohort in rural South Africa
Source: PLoS One. 2018 Aug 1;13(8):e0201445. doi: 10.1371/journal.pone.0201445 (PMC6070310; doi:10.1371/journal.pone.0201445)
Supplement: S1 Table — (DOCX) [file pone.0201445.s001.docx]

**Supplemental Table 1.** Logistic regression sensitivity analysis of the relationship between circumcision status and HIV status among HAALSI men with laboratory-confirmed HIV status (n=1945)

|  | **N** | **# HIV+** | **OR (95% CI)** | **p** | **aOR^1^ (95% CI)** | **p** |
| --- | --- | --- | --- | --- | --- | --- |
| **Circumcision status** |  |  |  |  |  |  |
| Circumcised | 497 | 113 | 0.93 (0.73, 1.19) | 0.6 | 0.87 (0.65, 1.16) | 0.3 |
| Uncircumcised | 1448 | 347 | 1 |  | 1 |  |
| **Circumcision type** |  |  |  |  |  |  |
| Hospital-based circumcision | 219 | 67 | 1.40 (1.02, 1.91) | 0.03 | 1.13 (0.78, 1.64) | 0.5 |
| Traditional initiation-based circumcision | 277 | 45 | 0.62 (0.44, 0.87) | 0.005 | 0.65 (0.43, 0.96) | 0.03 |
| No circumcision | 1448 | 347 | 1 |  | 1 |  |
| **Age at circumcision** |  |  |  |  |  |  |
| 1-13 | 112 | 26 | 0.96 (0.61, 1.51) | 0.9 | 0.84 (0.49, 1.43) | 0.5 |
| 14-18 | 181 | 33 | 0.71 (0.48, 1.05) | 0.09 | 0.66 (0.42, 1.06) | 0.09 |
| >18 | 202 | 54 | 1.16 (0.83, 1.62) | 0.4 | 1.08 (0.73, 1.59) | 0.7 |
| No circumcision | 1448 | 347 | 1 |  | 1 |  |
| **Age at circumcision among hospital-based circumcisions** |  |  |  |  |  |  |
| 1-13 | 34 | 10 | 1 |  | * |  |
| 14-18 | 55 | 16 | 0.98 (0.38, 2.52) | 1.0 | * |  |
| >18 | 128 | 41 | 1.13 (0.50, 2.58) | 0.8 | * |  |
| **Age at circumcision among traditional initiation-based circumcisions** |  |  |  |  |  |  |
| 1-13 | 77 | 15 | 1 |  | * |  |
| 14-18 | 126 | 17 | 0.64 (0.30, 1.38) | 0.3 | * |  |
| >18 | 74 | 13 | 0.88 (0.39, 2.01) | 0.8 | * |  |

^1^Adjusted for age (coded linearly in years), socio-economic quintiles, religion (African traditional vs. not), marital status (currently married vs. not), country of origin, education (any formal education vs. none), and number of lifetime sex partners (coded as 0-1, 2-4, or 5+)

*Adjusted analyses not conducted for age at circumcision stratified by circumcision type because data too sparse to support the models (i.e. the number of total HIV outcome events was less than 10 per predictor variable to be included in the adjusted model).
